# Synergistic role of circulating CD14++CD16+ monocytes and fibrinogen in predicting the cardiovascular events after myocardial infarction

**DOI:** 10.1002/clc.24005

**Published:** 2023-03-22

**Authors:** Chong Zhang, Shan Zeng, Wenjie Ji, Zhi Li, Haonan Sun, Tianming Teng, Ying Yu, Xin Zhou, Qing Yang

**Affiliations:** ^1^ Department of Cardiology Tianjin Medical University General Hospital Tianjin China; ^2^ Tianjin Key Laboratory of Cardiovascular Remodeling and Target Organ Injury, Heart Center, Pingjin Hospital Tianjin China; ^3^ Department of Pharmacology Tianjin Key Laboratory of Inflammatory Biology, Key Laboratory of Immune Microenvironment and Disease (Ministry of Education), Center for Cardiovascular Diseases, The Province and Ministry Co‐sponsored Collaborative Innovation Center for Medical Epigenetics, School of Basic Medical Sciences, Tianjin Medical University Tianjin China

**Keywords:** fibrinogen, major adverse cardiovascular events, monocyte subsets, myocardial infarction

## Abstract

**Background:**

Monocytes and fibrinogen (FIB) play important roles in driving acute and reparative inflammatory pathways after myocardial infarction (MI). In humans, there are three subsets of monocytes, namely, CD14++CD16− (Mon1), CD14++CD16+ (Mon2), and CD14+CD16++ (Mon3). During the inflammatory response, monocyte subsets express high levels of integrin α_M_β_2_ and protease‐activated receptors 1 and 3 to interact with FIB.

**Hypothesis:**

However, whether there is a synergistic role of FIB combined with Mon2 counts in prioritizing patients at high risk of future major adverse cardiovascular events (MACEs) after MI remains unknown.

**Methods:**

The MI patients who treated with primary percutaneous coronary intervention were enrolled. MI patients were categorized into four groups, that is, low FIB/low Mon2, low FIB/high Mon2, high FIB/low Mon2, and high FIB/high Mon2, according to cutoff values of 3.28 g/L for FIB and 32.20 cells/μL for Mon2. Kaplan−Meier survival analysis and Cox proportional hazards models were used to estimate the risk of MACEs of MI patients during a median follow‐up of 2.7 years. Mediating effects of high FIB levels and MACEs associated with high monocyte subsets were calculated by mediation analysis.

**Results:**

High FIB/high Mon2 group had the highest risk of MACEs during a median follow‐up of 2.7 years. Moreover, mediation analysis showed that a high FIB level could explain 24.9% (*p* < .05) of the increased risk of MACEs associated with Mon2.

**Conclusion:**

This work provides evidence indicating the translational potential of a synergistic role of FIB combined with Mon2 in prioritizing patients at high risk of future MACEs after MI.

## Graphical abstract

Association between the synergistic effect of FIR combined with Mon2 and future MACEs after STEMI. ACME, average causal mediating effect; ADE, average direct effect; CI, confidence interval; FIB, fibrinogen; HR, hazard ratio; MACEs, major adverse cardiovascular events; Mon2, CD14++CD16+ monocytes; Path A, the effect of Mon2 on MACEs; Path B, the effect of FIB on MACEs; Path C, the total effect of Mon2 on MACEs; Path C,' the direct effect of Mon2 on MACEs after controlling FIB; PPCI, primary percutaneous coronary intervention; STEMI, ST elevation myocardial infarction; TE, total effect. 

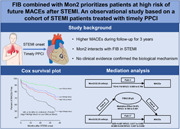

## 1 INTRODUCTION

Fibrinogen (FIB) plays a key role in promoting fibrin formation and dissolution in tissue remodeling and inflammation.^1^ Numerous clinical studies have also suggested that a high FIB level is associated with major adverse cardiovascular events (MACEs).^2^, ^3^, ^4^, ^5^, ^6^, ^7^ Moreover, basic research has illustrated a crucial role of FIB and its derivatives in driving inflammation,^8^ stimulating the release of monocyte chemoattractant protein‐1^9^ and promoting macrophage and monocyte adhesion in the inflammatory response.^10^

In humans, there are three subsets of monocytes, namely, CD14++CD16− (Mon1), CD14++CD16+ (Mon2), and CD14+CD16++ (Mon3)^11^ and high Mon2 counts were associated with MACEs in myocardial infarction (MI) patients.^12^, ^13^ Our previous studies examined Mon2 counts on Day 2 after ST elevation myocardial infarction (STEMI) onset^14^, ^15^ and changes of Mon2 counts trajectories in STEMI^16^ were associated with MACEs. Notably, monocyte subsets express high levels of integrin α_M_β_2_ ^17^ and protease‐activated receptors 1 and 3^18^ (PAR1 and PAR3) to interact with FIB in a pathway that involves inflammation‐driven coagulation activity and coagulation‐driven inflammation. To our knowledge, whether there is a synergistic role of FIB combined with Mon2 counts in prioritizing patients at high risk of future cardiovascular events after STEMI remains unknown.

In this study, considering the synergistic effect between FIB and monocytes, we hypothesized that the combined use of FIB and monocytes may be helpful for the risk stratification of MI patients presenting with high FIB levels.

## 2 MATERIALS AND METHODS

### 2.1 Study cohort

Our previous study^19^ described the two STEMI cohorts from Pingjin Hospital Heart Center. From November 2012 to May 2013, one cohort of 100 de novo STEMI patients treated with primary percutaneous coronary intervention (PPCI) within 12 h of symptom onset. From January 2015 to November 2015, another cohort of 133 de novo STEMI patients treated with timely PPCI. Our previous studies showed that the peak of Mon2 counts appeared on Day 2 after the onset of STEMI,^14^, ^16^ so we only measured circulating monocyte subsets counts on Day 2. We diagnosed and managed STEMI patients in accordance with the 2012 European Society of Cardiology guidelines.^20^ The detailed exclusion criteria were in accordance with our previous study,^19^ patients with the following conditions were excluded: (1) acute infections, cancer, previous MI, and decompensated heart failure in the past 6 months; (2) not suitable for PPCI; (3) multivessel coronary heart disease (CHD) with a planned PCI after discharge. Only the culprit vessel was treated during PPCI.

### 2.2 Flow cytometry analysis

Our previous study described the method used for monocyte subset flow cytometry analysis.^19^, ^21^ The method of angiographic analysis was detailed in the Supporting Information: Methods.

### 2.3 Clinical evaluations

All patients underwent transthoracic echocardiography (Philips iE33 System) on Day 2. We also included clinical data from routine blood and biochemical tests on Day 1 of STEMI onset.

### 2.4 Follow‐up

Follow‐up procedure described by our previous study.^19^ We followed‐up all participants routinely through outpatient clinic, telephone, or readmission records after discharge. The primary endpoint was defined as the occurrence of a first MACEs, including cardiogenic death, recurrent MI, nonfatal ischemic stroke, the need for emergency or elective repeat revascularization, and rehospitalization due to heart failure. With regard to cardiogenic death, if a noncardiovascular cause could be excluded, the deaths would be attributed to cardiogenic death.

### 2.5 Statistical analysis

STEMI patients were categorized into four groups, that is, low FIB/low Mon2, low FIB/high Mon2, high FIB/low Mon2, and high FIB/high Mon2, according to the optimal cutoff value of Mon2 (<32.20 or ≥32.20 cells/μL) and the optimal cutoff value of the FIB level (<3.28 or ≥3.28 g/L) calculated by receiver operator characteristic (ROC) curve analyses. Continuous variables with a normal distribution are expressed as the mean ± standard deviation. Nonparametric continuous variables were expressed as medians and 25th and 75th percentiles. Categorical variables were expressed as counts and percentages. Unpaired *t*‐test, Mann−Whitney *U* test, and *χ* ^2^ test were used to test for differences between groups for continuous and categorical variables, respectively. Covariates for the adjustment model included age, left ventricular ejection fraction (LVEF), creatinine, infarction location, and glucose level. We have tested the association between FIB levels and Mon2 levels by Spearman's correlation analysis and multiple linear regression after logarithmic transformation. ROC curve analyses were used to determine the optimal cutoff values of monocyte subset counts and covariates for the adjustment model for the prediction of MACEs. Kaplan−Meier survival analysis and Cox proportional hazards models were used to estimate the cumulative MACEs‐free rate based on the FIB level stratified by monocyte subset counts according to the optimal cutoff values of monocyte subsets and the optimal cutoff value of the FIB level. The low FIB/low monocyte subset group was used as a reference in Cox proportional hazards models. Mediating effects of high FIB levels and MACEs associated with high monocyte subsets were calculated by mediation analysis, and we used STATA command “medeff” to perform it. We used STATA 15.1 (STATA Corp.) for all analyses. A two‐tailed *p* < .05 was considered statistically significant.

## 3 RESULTS

### 3.1 Patient characteristics

Two hundred and thirty‐three STEMI patients enrolled in this study. In this study, 12 cases were lost to follow‐up, and 1 case died on Day 2 of STEMI onset. Finally, 220 cases included in this study. The patient characteristics of STEMI patients categorized by Mon2 combined with FIB are shown in Table 1. Compared with the low FIB/low Mon2 group, the high FIB/high Mon2 group was more likely to have a higher creatinine level, higher glucose level, higher total monocyte counts, and monocyte subset counts. In outcomes, high FIB/high Mon2 group have a higher percentage of patients with MACEs and hospitalization for heart failure (HHF), as shown in Supporting Information: Table 3. Compared with the non‐MACEs group, the MACEs group was more likely to have older individuals and those with higher glucose levels, higher FIB levels, more anterior wall MI, higher total monocyte counts, higher Mon1 counts, higher Mon2 counts, and lower LVEF, as shown in Supporting Information: Table 1.

**Table 1:** Baseline clinical features of STEMI patients categorized by Mon2 combined with FIB.

	Low FIB/low Mon2 (*n* = 82)	Low FIB/high Mon2 (*n* = 35)	High FIB/low Mon2 (*n* = 55)	High FIB/high Mon2 (*n* = 48)	*p* Value
Demographics					
Age (year)	58.63 ± 10.81	59.83 ± 12.18	64.82 ± 11.54	62.23 ± 12.09	.785
Sex, male, *n* (%)	73 (89.0)	30 (85.7)	39 (70.9)	34 (70.8)	.017
Body mass index (kg/m)^2^	24.92 ± 2.95	24.30 ± 3.43	24.99 ± 3.46	25.44 ± 4.21	.049
History					
Smoking, *n* (%)	56 (68.3)	23 (65.7)	32 (58.2)	31 (64.6)	.683
Hypertension, *n* (%)	36 (43.9)	23 (65.7)	28 (50.9)	30 (62.5)	.077
Diabetes, *n* (%)	14 (17.1)	8 (22.9)	12 (21.8)	12 (25.0)	.724
Clinical parameters					
Infarct location, anterior wall (%)	37 (45.1)	18 (51.4)	27 (49.1)	26 (54.2)	.78
Symptom to admission time (h)	3.0 (1.5−4.5)	4.0 (2.0−8.0)	2.0 (1.3−3.8)	3.8 (2.0−6.0)	.006
Creatinine (μmol/L)	70.61 ± 10.94	72.23 ± 18.79	71.76 ± 22.01	83.25 ± 46.63	<.001
Fibrinogen (g/L)	2.84 (2.52−3.08)	2.93 (2.52−3.15)	3.68 (3.43−4.50)	3.91 (3.51−4.45)	<.001
Glucose (mmol/L)	7.40 (6.00−9.60)	6.20 (5.40−8.30)	7.00 (6.40−9.80)	6.90 (5.70−8.80)	.046
Low density lipoprotein (mmol/L)	2.87 (2.13−3.31)	2.30 (2.18−2.83)	3.00 (2.43−3.66)	2.57 (1.99−3.02)	.006
High density lipoprotein (mmol/L)	0.99 (0.84−1.17)	1.20 (1.06−1.30)	1.02 (0.83−1.13)	1.05 (0.90−1.28)	.001
Triglycerides (mmol/L)	1.70 (1.22−2.11)	1.71 (0.99−2.29)	1.40 (1.05−1.91)	1.58 (1.03−2.49)	.39
LVEF (%)	51 (43−55)	48 (45−54)	52 (45−55)	48 (40−55)	.485
Monocytes on Day 2					
Total monocytes (cells/μL)	460.82 (343.45−600.99)	671.49 (469.04−770.83)	469.56 (313.25−577.43)	587.07 (397.34−978.62)	<.001
Mon1 (cells/μL)	333.90 (243.29−425.10)	487.25 (362.93−603.06)	360.00 (245.13−426.33)	446.01 (308.24−697.72)	<.001
Mon2 (cells/μL)	16.71 (10.71−21.47)	55.42 (43.48−87.08)	16.34 (10.48−22.81)	58.87 (44.60−105.68)	<.001
Mon3 (cells/μL)	23.25 (13.83−35.36)	32.58 (21.77−52.34)	25.91 (17.29−42.58)	42.11 (29.69−57.90)	<.001

Abbreviations: FIB, fibrinogen; HHF, hospitalization for heart failure; LVEF, left ventricular ejection fraction; MACEs, major adverse cardiovascular events; MI, myocardial infarction; Mon1, CD14++CD16, monocytes; Mon2, CD14++CD16+ monocytes; Mon3, CD14+CD16++ monocytes; STEMI, ST elevation myocardial infarction.

### 3.2 Associations of FIB, Mon2 levels, and MACEs during follow‐up

**Figure 1:**
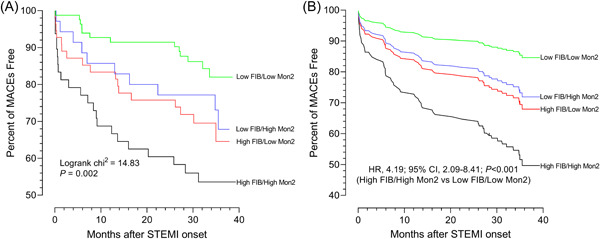
Synergistic effect of Mon2 combined with FIB in predicting cardiovascular events after myocardial infarction. (A) Kaplan−Meier survival analysis stratified by categories according to FIB and Mon2 levels. (B) Survival curves stratified by categories according to FIB and Mon2 levels in the Cox proportional hazards model after adjustment for age, LVEF, creatinine, infarction location, and glucose level based on the optimal cutoff values derived from ROC curve analyses.  FIB, fibrinogen; LVEF, left ventricular ejection fraction; MACEs, major adverse cardiovascular events; Mon2, CD14++CD16+ monocytes; ROC, receiver operator characteristic; STEMI, ST elevation myocardial infarction.

Spearman's correlation analysis showed the positive association between Mon2 levels and FIB, and the correlation coefficient was 0.179 (*p* = .008). Moreover, in multiple linear regression, high Mon2 levels were associated with high FIB (adjusted coefficient: 0.042, 95% CI: 0.003−0.081, *p* = .037) (Supporting Information: Table 6). After stratifying FIB by Mon2 levels, the incidence rates of MACEs increased greatly among the four groups in the order from low FIB/low Mon2, low FIB/high Mon2, high FIB/low Mon2, to high FIB/high Mon2 (*p* < .05). The Kaplan−Meier survival analysis (Figure 1A) and Cox survival plot adjusted by potential covariates (Figure 1B) indicated that the high FIB/high Mon2 group had the highest risk of MACEs compared with the low FIB/low Mon2 group. We also found high FIB/high Mon2 group showed the highest risk of HHF compared with the low FIB/low Mon2 group (adjusted HR: 9.78, 95% CI: 2.57−37.18, *p* = .001). According to the optimal cutoff values derived from ROC curve analyses (Supporting Information: Table 2), synergistic effects were also observed with FIB stratified by Mon1, Mon3, and total monocytes with MACEs. Moreover, FIB, Mon1, and Mon2 were independent risk factors for MACEs (Table 2). Of note, we have tested various cutoff values of FIB in the Cox multivariate model, and we also found there remain a significant association between the synergistic role of FIB combined with Mon2 counts and MACEs among different cutoff values (3.4 and 2.8 g/L) mentioned in literatures^3^, ^22^ (Supporting Information: Tables 4 and 5). In addition, we found LVEF, glucose were independent risk factors for MACEs in multivariable Cox proportional hazard models (Supporting Information: Table 7).

**Table 2:** Cox proportional hazard models for the synergistic effect between admission FIB and monocyte subsets and MACEs during follow‐up.

Group	Crude model		Adjust model^a^	
	HR and 95% CI	*p* Value	HR and 95% CI	*p* Value
FIB (≥3.28 g/L)	2.28 (1.36−3.82)	.002	2.54 (1.49−4.31)	.001
Total monocytes and monocyte subsets				
Total monocytes (≥490.11 cells/μL)	1.71 (1.02−2.87)	.042	1.53 (0.91−2.59)	.112
Mon1 (≥371.17 cells/μL)	2.08 (1.24−3.48)	.006	2.03 (1.20 −3.42)	.008
Mon2 (≥32.20 cells/μL)	1.89 (1.15−3.11)	.013	2.13 (1.28−3.53)	.003
Mon3 (≥35.62 cells/μL)	1.15 (0.69−1.90)	.587	1.11 (0.66−1.86)	.693
Fibrinogen stratified by total monocytes				
Low FIB/low monocyte (reference)	NA	NA	NA	NA
Low FIB/high monocyte	1.48 (0.64−3.42)	.36	1.31 (0.56−3.04)	.536
High FIB/low monocyte	1.95 (0.84−4.50)	.118	2.07 (0.88−4.86)	.095
High FIB/high monocyte	3.83 (1.78−8.23)	.001	3.67 (1.68−8.01)	.001
Fibrinogen stratified by Mon1				
Low FIB/low Mon1 (reference)	NA	NA	NA	NA
Low FIB/high Mon1	1.50 (0.66−3.42)	.335	1.40 (0.61−3.20)	.431
High FIB/low Mon1	1.64 (0.72−3.74)	.24	1.67 (0.72−3.87)	.233
High FIB/high Mon1	4.39 (2.11−9.13)	<.001	4.58 (2.18−9.61)	<.001
Fibrinogen stratified by Mon2				
Low FIB/low Mon2 (reference)	NA	NA	NA	NA
Low FIB/high Mon2	1.83 (0.80−4.18)	.151	1.98 (0.86−4.54)	.108
High FIB/low Mon2	2.24 (1.09−4.62)	.028	2.32 (1.10−4.87)	.027
High FIB/high Mon2	3.57 (1.80−7.10)	<.001	4.19 (2.09−8.41)	<.001
Fibrinogen stratified by Mon3				
Low FIB/low Mon3 (reference)	NA	NA	NA	NA
Low FIB/high Mon3	1.73 (0.64−4.65)	.28	1.56 (0.57−4.29)	.384
High FIB/low Mon3	2.95 (1.10−7.95)	.032	3.27 (1.20−8.94)	.021
High FIB/high Mon3	3.91 (1.47−10.36)	.006	3.53 (1.32−9.47)	.012

Abbreviations: CI, confidence interval; FIB, fibrinogen; HR, hazard ratio; MACEs, major adverse cardiovascular events; Mon1, CD14++CD16− monocytes; Mon2, CD14++CD16+ monocytes; Mon3, CD14+CD16++ monocytes.

^a^ Adjusted for age, left ventricular ejection fraction, creatinine, infarction location, and glucose level.

### 3.3 Mediating effect of high FIB levels and MACEs associated with high Mon2 levels

**Figure 2:**
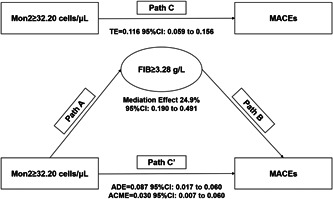
Mediating effect of high FIB levels and MACEs associated with high Mon2 levels. The mediation analysis shows that high FIB levels could explain 24.9% (*p* < .05) of the increased risk of MACEs associated with high Mon2 levels after adjustment for age, LVEF, creatinine, infarction location, and glucose level based on the optimal cutoff values derived from ROC curve analyses. ACME, average causal mediating effect; ADE, average direct effect; FIB, fibrinogen; LVEF, left ventricular ejection fraction; MACEs, major adverse cardiovascular events; Mon2, CD14++CD16+ monocytes; Path A, the effect of Mon2 on MACEs; Path B, the effect of FIB on MACEs; Path C, the total effect of Mon2 on MACEs; Path C', the direct effect of Mon2 on MACEs after controlling FIB; ROC, receiver operator characteristic; TE, total effect.

The mediation analysis was performed with the FIB level and monocyte subsets as dichotomic variables by using the optimal cutoff values derived from ROC curve analyses. Univariate mediation analysis showed that a high FIB level could explain 13.7% (*p* < .05) of the increased risk of MACEs associated with high Mon2 levels. After multivariate adjustment, a high FIB level could explain 24.9% (*p* < .05) of the increased risk of MACEs associated with high Mon2 levels (Figure 2). The mediation analysis showed a synergistic role of FIB combined with Mon2 counts in prioritizing patients at high risk of MACEs after STEMI that reflected the baseline clinical features of STEMI patients categorized by Mon2 combined with FIB. Total monocytes, Mon1 and Mon3 showed no mediating effects with FIB (Supporting Information: Figure 1).

## 4 DISCUSSION

In this study, STEMI patients were treated with timely PPCI, and we found that FIB levels were positively correlated with Mon2 levels. In the four mutually exclusive groups categorized by FIB level (<3.28 and ≥3.28 g/L) and monocyte subsets, we found synergistic effects of monocyte subsets and FIB with MACEs during follow‐up. Moreover, high FIB levels had a significant mediating effect on MACEs associated with high Mon2. FIB combined with Mon2 may be helpful for risk stratification of STEMI patients. Notably, in this study, we also found a synergistic role between FIB and Mon2‐directed inflammation in STEMI and MI remodeling.

FIB is a coagulation protein in the blood as well as a significant marker of inflammatory response.^23^ FIB is now deemed to play a key role in the acute phase response caused by tissue injury and remodeling and repairing damaged tissue in second phase.^1^ Moreover, FIB has been demonstrated to be a risk factor for MACEs in healthy adults and CHD patients. According to a meta‐analysis including individual participant data from 52 cohort studies, the baseline level of FIB in people without known CVD was an independent risk factor for MACEs over a period of 10 years.^7^ Additionally, numerous studies have demonstrated that FIB levels are related to short‐term and long‐term MACEs in CHD patients.^3^, ^4^, ^5^, ^6^ However, the association between FIB levels and MACEs remains controversial. The Prospective Epidemiological Study of Myocardial Infarction (PRIME)^24^, ^25^ included middle‐aged men without CHD and showed that the association of FIB levels and MACEs was rescinded after adjustment for traditional risk factors, which is consistent with the Atherosclerosis Risk in Communities study.^26^ A prospective cohort study included 766 participants with or without CVD and a median of 8.7 years of follow‐up. Therefore, the association between FIB and MACEs should be demonstrated by further clinical studies. Moreover, few studies have evaluated the association between the FIB level and the long‐term prognosis of STEMI patients after PCI. In the present study, we found that FIB levels were significantly associated with MACEs in STEMI patients after PCI during follow‐up. The results were consistent with previous studies,^2^, ^3^, ^4^, ^5^, ^6^, ^7^ indicating that high FIB level played a key role in STEMI. The present study also demonstrated a positive association between Mon2 and FIB levels, and FIB levels had a significant mediating effect on MACEs associated with Mon2. Biological mechanisms between FIB and Mon2 may help explain these findings. First, human monocytes express high levels of integrin α_M_β_2_,^17^ and among human monocyte subsets, Mon1 and Mon2 express similarly high levels of integrin α_M_, while Mon3 expresses no or very low levels of integrin α_M_.^27^ Mon1 expresses higher levels of integrin β2 than those of Mon2 and Mon3.^27^ Integrin α_M_β_2_ mediates the attachment of monocytes and FIB to participate in the inflammatory response.^17^ Moreover, some factors, such as FXIII, can stabilize fibrin clots through crosslinking fibrin polymers,^28^ which are important in modifying inflammation. FXIII is highly expressed in monocytes^29^ and crosslinking of AT1 receptors results in the enhancement of adhesiveness to endothelial cells.^30^ Therefore, FXIII can influence inflammation by mediating the attachment of monocytes and FIB functions. Second, during inflammatory processes, there is a reciprocal pathway that involves inflammation‐driven coagulation activity and coagulation‐driven inflammation.^1^ Thaler et al. found that Mon2 expressed more PAR1 and PAR3 than those of other monocyte subsets, which contributed to a procoagulant milieu through the induction of PAI‐1, and a procoagulatory environment could in turn activate monocytes through the interaction of thrombin with PAR1 and PAR3,^18^ which was inseparable from the contributions made by FIB, an important downstream target of thrombin.^1^ Third, in the acute phase response leading to extensive fibrin deposition, inappropriate FIB coagulation‐mediated activation by inflammation can be detrimental to tissue repair and may result in early complications and myocardial fibrosis.^31^ Studies have demonstrated that the proinflammatory properties of Mon2^32^, ^33^, ^34^ are closely related to atherosclerosis and post‐MI healing, which are involved in the FIB‐mediated myocardial repair process. Moreover, the prognostic value of Mon1 could be partly explained by the rapid transformation from Mon1 to Mon2 during STEMI.^35^ In this study, we confirmed that a high FIB level could explain 24.9% (*p* < .05) of the increased risk for MACEs associated with a high Mon2 level, which further supports the synergistic effect between Mon2 and FIB during the acute phase response and long‐term prognosis of STEMI patients. Although total monocytes, Mon1 and Mon3 showed no mediating effect with FIB, the multivariate Cox proportional hazards model showed a synergistic effect of FIB with total monocytes, Mon1 and Mon3 on driving inflammation in STEMI and long‐term prognosis. In the multivariate‐adjusted Cox proportional hazards model, we also identified some risk factors associated with MACEs in our study, such as LVEF, glucose level, which indicated the complex biological mechanisms during STEMI. Future work is urgently needed to investigate the underlying mechanisms among monocytes and FIB as well as other risk factors.

Several limitations should be acknowledged in this study. First, due to the small sample size and limited numbers of MACEs, to avoid the potential issues of overfitting and to maintain the model's parsimony, we only included age, LVEF, creatinine, infarction location, and glucose level for covariate adjustment, which were statistically different between patients with and without MACEs. Other risk factors, such as sex, symptom to admission time, and underlying cardiovascular diseases were not included for adjustment. Second, our study was based on the Chinese population who had different physical conditions and lifestyles from the Western population. Third, in addition to monocyte subsets, other biomarkers, such as interleukin‐1β and interleukin‐6 levels, which were closely related to STEMI, were not included in our study.

In conclusion, in a cohort of STEMI patients treated with timely PPCI, we demonstrated the translational potential of a synergistic role of FIB combined with Mon2 counts in prioritizing patients at high risk of future cardiovascular events. Further works are warranted to validate our findings.

## CONFLICT OF INTEREST STATEMENT

The authors declare no conflict of interest.

## Supporting information

Supplementary information.

## Data Availability

The data that support the findings of this study are available from the corresponding author.
