# Supplementary material for: Synergistic role of circulating CD14++CD16+ monocytes and fibrinogen in predicting the cardiovascular events after myocardial infarction
Source: Clin Cardiol. 2023 Mar 22;46(5):521–8. doi: 10.1002/clc.24005 (PMC10189084; doi:10.1002/clc.24005)
Supplement: Supplementary file 1 — Supplementary information. [file CLC-46-521-s001.docx]

**Supplemental Material**

**Synergistic Role of Circulating** **CD14++CD16+ Monocytes and** **Fibrinogen in Predicting the Cardiovascular Events after Myocardial Infarction**

**List of Content**

1. **Supplemental Methods**

- **Flow cytometry analysis**

1. **Supplemental Figures**

- **sFigure 1.** The mediation analysis performed with FIB level and total monocytes, Mon1 and Mon3 respectively

1. **Supplemental Tables**

- **sTable 1.** Baseline clinical features of STEMI patients with and without MACEs
- **sTable 2.** Cutoff values of monocyte and adjusted variables
- **sTable 3.** Individual Outcomes of STEMI patients categorized by Mon2 combined with FIB
- **sTable 4.** Cox proportional hazard models for the association between FIB (cutoff value: 3.4 g/L) stratified by monocyte subsets and MACEs
- **sTable 5.** Cox proportional hazard models for the association between FIB (cutoff value: 2.8 g/L) stratified by monocyte subsets and MACEs
- **sTable 6.** Multiple linear regression for the association between Mon2 and FIB
- **sTable 7.** Multivariable Cox proportional hazard models for the relationship between the synergistic role of FIB combined with Mon2 counts and MACEs

**Supplemental Methods**

***Flow cytometry analysis***

The methods of flow cytometry analysis were described in our previous study [1, 2]. Fasting blood samples were collected through the antecubital vein with ethylenediaminetetraacetic acid as an anticoagulant. Then, 50 μL of blood sample was incubated with a mixture containing the following antibodies (10 μL each): fluorescein isothiocyanate (FITC)–labeled anti-human CD14 (clone M5E2), phycoerythrin (PE)-labeled anti-human CD16 (clone 3G8), PE-Cy5-labeled anti-human CD86 (clone IT2.2), and PE-Cy7 labeled antihuman CD41 (clone HIP8). After being kept at room temperature for 15 min, erythrocyte lysate was added for another 10 min. We used the following isotope controls: IgG2a-FITC (clone MOPC-173), IgG1-PE (clone MOPC-27), IgG2b-PE-Cy5 (clone MPC-11), and IgG1-PE-Cy7 (clone MPOC-21). All antibodies were purchased from BioLegend (San Diego, CA). The gating boundaries and compensations were set using the unstained, single-stained, and Fluorescence Minus One controls. For absolute cell counting, 50 μL of Flow-Count™ fluorescence microbeads (Beckman-Coulter, Miami, FL) was added. We used Cytomics FC500 (BeckmanCoulter, Miami, FL) and FlowJo software (Treestar, Ashland, OR) for data acquisition and analysis.

**References**

1. Dong S, Ji W, Zeng S, et al. Admission Low-Density Lipoprotein Cholesterol Stratified by Circulating CD14++CD16+ Monocytes and Risk for Recurrent Cardiovascular Events Following ST Elevation Myocardial Infarction: Lipid Paradox Revised. *J Cardiovasc Transl Res*. 2020;13(6):916-927.
2. Zhou X, Zhang L, Ji WJ, el al. Variation in dietary salt intake induces coordinated dynamics of monocyte subsets and monocyte-platelet aggregates in humans: implications in end organ inflammation. *PLoS One*. 2013;8(4):e60332


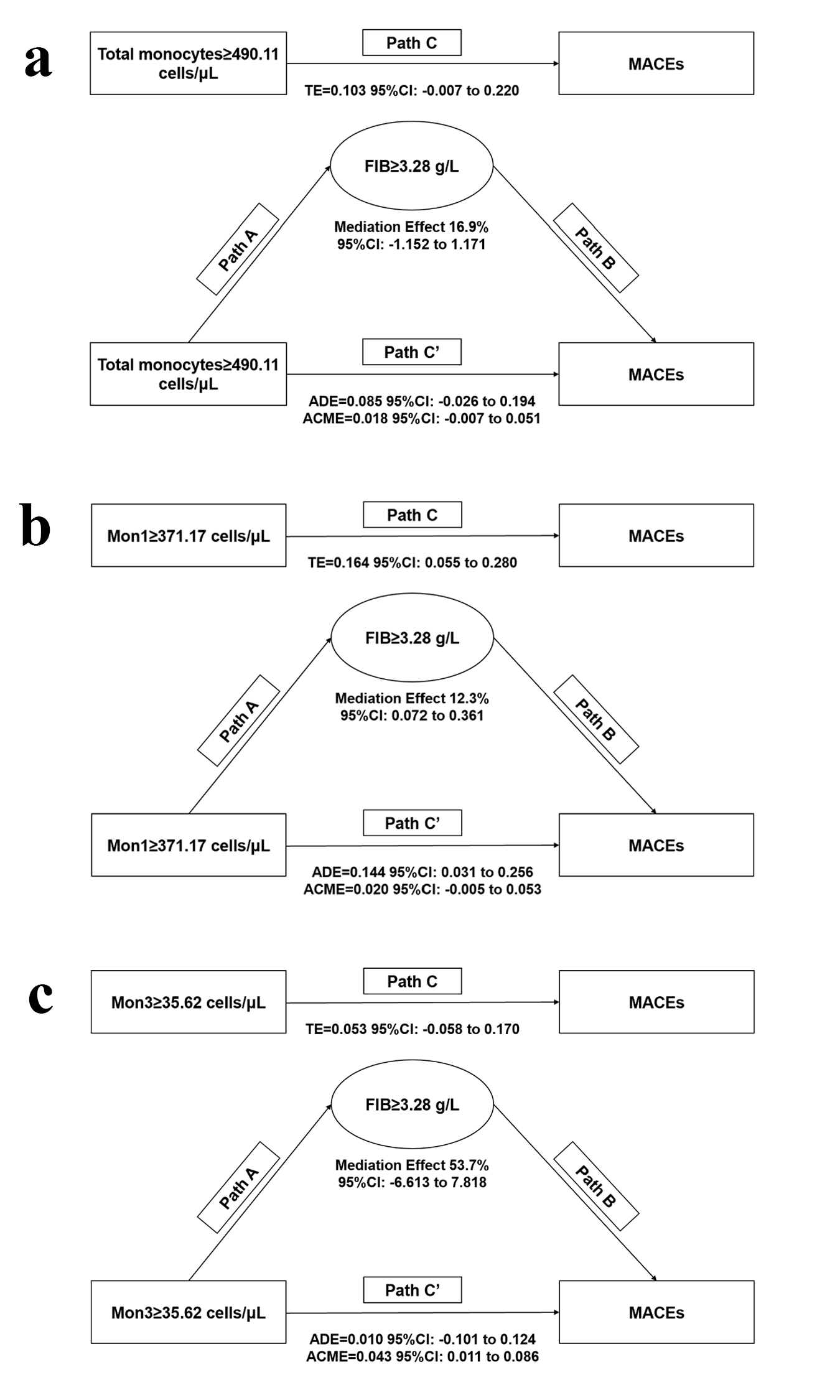


**sFigure 1****.** The mediation analysis performed with FIB level and total monocytes, Mon1 and Mon3 respectively. **a** Total monocytes showed that no mediating effect with FIB; **b** Mon1 showed that no mediating effect with FIB; **c** Mon3 showed that no mediating effect with FIB; The adjusted model included age, LVEF, creatinine, infarction location and glucose level were based on the optimal cutoff values derived from ROC curve analyses. **Abbreviations:** *Path A* the effect of total monocytes or monocyte subsets on MACEs, *Path B* the effect of FIB on MACEs, *Path C* the total effect of total monocytes or monocyte subsets on MACEs, *Path C'* the direct effect of total monocytes or monocyte subsets on MACEs after controlling FIB, *TE* total effect, *ACME* average causal mediating effect, *ADE* average direct effect, *FIB* fibrinogen, *LVEF* left ventricular ejection fraction, *MACEs* major adverse cardiovascular events, *Mon1* CD14++CD16- monocytes, *Mon2* CD14++CD16+ monocytes, *Mon3* CD14+CD16++ monocytes, *ROC* receiver operator characteristic.

**sTable 1.** Baseline clinical features of STEMI patients with and without MACEs

|  | MACEs (+) n=62 | MACEs (-) n=158 | *P* value |
| --- | --- | --- | --- |
| **Demographics** |  |  |  |
| Age, year | 63.42 ± 11.80 | 60.27 ± 11.57 | 0.072 |
| Sex, male, n (%) | 48 (77.4) | 128 (81.0) | 0.549 |
| Body mass index, kg/m^2^ | 24.43 ± 3.12 | 25.16 ± 3.56 | 0.156 |
| **History** |  |  |  |
| Smoking, n (%) | 40 (64.5) | 102 (64.6) | 0.995 |
| Hypertension, n (%) | 33 (53.2) | 84 (53.2) | 0.986 |
| Diabetes, n (%) | 16 (27.6) | 30 (19.0) | 0.263 |
| **Clinical parameters** |  |  |  |
| Infarct location, anterior wall (%) | 37 (59.7) | 71 (44.9) | 0.049 |
| Symptom to admission time, hours | 3.0 (2.0 to 5.0) | 3.0 (1.5 to 5.0) | 0.490 |
| Creatinine, μmol/L | 68 (56 to 77) | 70 (62 to 81) | 0.292 |
| Fibrinogen, g/L | 3.43 (2.99 to 4.20) | 3.19 (2.78 to 3.57) | 0.002 |
| Glucose, mmol/L | 7.65 (5.80 to 10.0) | 6.90 (6.00 to 8.60) | 0.099 |
| Low density lipoprotein, mmol/L | 2.67 (2.05 to 3.13) | 2.72 (2.21 to 3.34) | 0.174 |
| High density lipoprotein, mmol/L | 1.06 (0.91 to 1.26) | 1.04 (0.88 to 1.22) | 0.35 |
| Triglycerides, mmol/L | 1.59 (1.03 to 2.18) | 1.62 (1.11 to 2.13) | 0.784 |
| LVEF, % | 46 (38 to 52) | 52 (45 to 55) | <0.001 |
| **Circulating monocyte subsets on day 2** | |  |  |
| Total monocytes, cells/μL | 555.82 (412.73 to 716.11) | 486.16 (357.96 to 662.30) | 0.048 |
| Mon1, cells/μL | 400.59 (312.49 to 559.05) | 360.07 (248.48 to 487.25) | 0.026 |
| Mon2, cells/μL | 32.84 (16.71 to 69.69) | 22.00 (12.25 to 43.48) | 0.004 |
| Mon3, cells/μL | 26.75 (17.64 to 43.88) | 28.79 (18.35 to 45.24) | 0.716 |

Abbreviations: *LVEF* left ventricular ejection fraction, *MACEs* major adverse cardiovascular events, *Mon1* CD14++CD16- monocytes, *Mon2* CD14++CD16+ monocytes, *Mon3* CD14+CD16++ monocytes.

**sTable 2.** Cutoff values of monocyte and adjusted variables

|  | **Cutoff values** | **Sensitivity (%)** | **Specificity (%)** |  |
| --- | --- | --- | --- | --- |
|  |  |  |  |  |
| **Monocyte** | |  |  |  |
| Total monocytes, cells/μL | 490.11 | 63 | 51 |  |
| Mon1, cells/μL | 371.17 | 63 | 58 |  |
| Mon2, cells/μL | 32.2 | 52 | 68 |  |
| Mon3, cells/μL | 35.62 | 42 | 63 |  |
| **Adjusted variables** | |  |  |  |
| Age, year | 61 | 60 | 52 |  |
| Glucose, mmol/L | 7.4 | 55 | 60 |  |
| LVEF, % | 52 | 31 | 47 |  |
| Creatinine, μmol/L | 72 | 39 | 56 |  |
| Fibrinogen, g/L | 3.28 | 63 | 59 |  |

Note: Cutoff values of monocyte and adjusted variables were derived from ROC curve analyses.

Abbreviations: *LVEF* left ventricular ejection fraction, *Mon1* CD14++CD16- monocytes, *Mon2* CD14++CD16+ monocytes, *Mon3* CD14+CD16++ monocytes, *ROC* receiver operator characteristic.

**sTable 3.** Individual Outcomes of STEMI patients categorized by Mon2 combined with FIB

|  | Low FIB/low Mon2 n=82 | Low FIB/high Mon2 n=35 | High FIB/low Mon2 n=55 | High FIB/high Mon2 n=48 | *P* value |
| --- | --- | --- | --- | --- | --- |
| MACEs, n (%) | 13 (15.9) | 10 (28.6) | 17 (30.9) | 22 (45.8) | 0.003 |
| Cardiogenic death, n (%) | 1 (1.2) | 2 (5.7) | 5 (9.1) | 5 (10.4) | 0.11 |
| HHF, n (%) | 3 (3.7) | 1 (2.9) | 2 (3.6) | 9 (18.8) | 0.003 |
| Nonfatal ischemic stroke, n (%) | 1 (1.2) | 1 (2.9) | 0 (0) | 2 (4.2) | 0.41 |
| Revascularization, n (%) | 3 (3.7) | 6 (17.1) | 5 (9.1) | 4 (8.3) | 0.11 |
| Recurrent MI, n (%) | 5 (6.1) | 0 (0) | 5 (9.1) | 2 (4.2) | 0.30 |

Abbreviations: *FIB* fibrinogen, *Mon2* CD14++CD16+ monocytes, *MACEs* major adverse cardiovascular events, *HHF* hospitalization for heart failure, *MI* myocardial infarction.

**sTable 4.** Cox proportional hazard models for the association between FIB (cutoff value: 3.4 g/L) stratified by monocyte subsets and MACEs

| Group | Crude model | |  | | Adjust model ^a^ | | | |
| --- | --- | --- | --- | --- | --- | --- | --- | --- |
|  | HR and 95%CI | *P* value |  | | HR and 95%CI | | *P* value | |
| Fibrinogen stratified by total monocytes | |  |  |  | |  | |  |
| Low FIB/low monocyte (reference) | N. A. | N. A. |  |  | | N. A. | | N. A. |
| Low FIB/high monocyte | 1.50 (0.71 to 3.19) | 0.288 |  |  | | 1.34 (0.63 to 2.86) | | 0.454 |
| High FIB/low monocyte | 1.72 (0.76 to 3.90) | 0.195 |  |  | | 1.80 (0.79 to 4.12) | | 0.165 |
| High FIB/high monocyte | 3.62 (1.74 to 7.53) | 0.001 |  |  | | 3.43 (1.62 to 7.23) | | 0.001 |
| Fibrinogen stratified by Mon1 |  |  |  |  | |  | |  |
| Low FIB/low Mon1 (reference) | N. A. | N. A. |  |  | | N. A. | | N. A. |
| Low FIB/high Mon1 | 1.55 (0.74 to 3.24) | 0.248 |  |  | | 1.45 (0.69 to 3.04) | | 0.331 |
| High FIB/low Mon1 | 1.42 (0.63 to 3.23) | 0.397 |  |  | | 1.44 (0.63 to 3.29) | | 0.394 |
| High FIB/high Mon1 | 4.34 (2.14 to 8.80) | <0.001 |  |  | | 4.46 (2.17 to 9.14) | | <0.001 |
| Fibrinogen stratified by Mon2 |  |  |  |  | |  | |  |
| Low FIB/low Mon2 (reference) | N. A. | N. A. |  |  | | N. A. | | N. A. |
| Low FIB/high Mon2 | 1.97 (0.95 to 4.08) | 0.069 |  |  | | 2.17 (1.04 to 4.52) | | 0.039 |
| High FIB/low Mon2 | 2.14 (1.05 to 4.38) | 0.037 |  |  | | 2.20 (1.05 to 4.62) | | 0.038 |
| High FIB/high Mon2 | 3.38 (1.70 to 6.72) | <0.001 |  |  | | 3.90 (1.94 to 7.80) | | <0.001 |
| Fibrinogen stratified by Mon3 |  |  |  |  | |  | |  |
| Low FIB/low Mon3 (reference) | N. A. | N. A. |  |  | | N. A. | | N. A. |
| Low FIB/high Mon3 | 0.60 (0.25 to 1.40) | 0.233 |  |  | | 0.65 (0.27 to 1.54) | | 0.327 |
| High FIB/low Mon3 | 1.36 (0.70 to 2.66) | 0.366 |  |  | | 1.61 (0.79 to 3.29) | | 0.189 |
| High FIB/high Mon3 | 2.23 (1.21 to 4.12) | 0.011 |  |  | | 2.21 (1.18 to 4.14) | | 0.014 |

Note: ^a^ Adjusted for age, left ventricular ejection fraction, creatinine, infarction location and glucose level.

Abbreviations: *CI* confidence interval, *FIB* fibrinogen, *HR* hazard ratio, *MACEs* major adverse cardiovascular events, *Mon1* CD14++CD16- monocytes, *Mon2* CD14++CD16+ monocytes, *Mon3* CD14+CD16++ monocytes.

**sTable 5.** Cox proportional hazard models for the association between FIB (cutoff value: 2.8 g/L) stratified by monocyte subsets and MACEs

| Group | Crude model | |  | | Adjust model ^a^ | | | |
| --- | --- | --- | --- | --- | --- | --- | --- | --- |
|  | HR and 95%CI | *P* value |  | | HR and 95%CI | | *P* value | |
| Fibrinogen stratified by total monocytes | |  |  |  | |  | |  |
| Low FIB/low monocyte (reference) | N. A. | N. A. |  |  | | N. A. | | N. A. |
| Low FIB/high monocyte | 1.21 (0.29 to 5.08) | 0.792 |  |  | | 1.18 (0.28 to 4.96) | | 0.821 |
| High FIB/low monocyte | 1.70 (0.51 to 5.73) | 0.390 |  |  | | 2.14 (0.62 to 7.36) | | 0.227 |
| High FIB/high monocyte | 3.24 (0.99 to 10.55) | 0.051 |  |  | | 3.54 (1.08 to 11.59) | | 0.037 |
| Fibrinogen stratified by Mon1 |  |  |  |  | |  | |  |
| Low FIB/low Mon1 (reference) | N. A. | N. A. |  |  | | N. A. | | N. A. |
| Low FIB/high Mon1 | 0.79 (0.20 to 3.16) | 0.737 |  |  | | 0.82 (0.20 to 3.29) | | 0.780 |
| High FIB/low Mon1 | 1.15 (0.39 to 3.37) | 0.805 |  |  | | 1.41 (0.47 to 4.23) | | 0.539 |
| High FIB/high Mon1 | 2.98 (1.06 to 8.40) | 0.038 |  |  | | 3.54 (1.25 to 10.05) | | 0.018 |
| Fibrinogen stratified by Mon2 |  |  |  |  | |  | |  |
| Low FIB/low Mon2 (reference) | N. A. | N. A. |  |  | | N. A. | | N. A. |
| Low FIB/high Mon2 | 3.54 (0.88 to 14.17) | 0.074 |  |  | | 4.63 (1.14 to 18.84) | | 0.032 |
| High FIB/low Mon2 | 2.74 (0.96 to 7.85) | 0.061 |  |  | | 3.44 (1.19 to 9.99) | | 0.023 |
| High FIB/high Mon2 | 4.32 (1.51 to 12.34) | 0.006 |  |  | | 5.75 (1.99 to 16.61) | | 0.001 |
| Fibrinogen stratified by Mon3 |  |  |  |  | |  | |  |
| Low FIB/low Mon3 (reference) | N. A. | N. A. |  |  | | N. A. | | N. A. |
| Low FIB/high Mon3 | 0.60 (0.12 to 2.99) | 0.537 |  |  | | 0.72 (0.14 to 3.59) | | 0.685 |
| High FIB/low Mon3 | 1.71 (0.71 to 4.11) | 0.231 |  |  | | 2.27 (0.91 to 5.62) | | 0.078 |
| High FIB/high Mon3 | 2.09 (0.85 to 5.12) | 0.106 |  |  | | 2.58 (1.03 to 6.46) | | 0.042 |

Note: ^a^ Adjusted for age, left ventricular ejection fraction, creatinine, infarction location and glucose level.

Abbreviations: *CI* confidence interval, *FIB* fibrinogen, *HR* hazard ratio, *MACEs* major adverse cardiovascular events, *Mon1* CD14++CD16- monocytes, *Mon2* CD14++CD16+ monocytes, *Mon3* CD14+CD16++ monocytes.

**sTable 6.** Multiple linear regression for the association between Mon2 and FIB

|  | Crude model | |  | | Adjust model ^a^ | | | |
| --- | --- | --- | --- | --- | --- | --- | --- | --- |
|  | Coef. and 95%CI | *P* value |  | | | Coef. and 95%CI | *P* value | |
| Mon2, (Log) | 0.040 (0.002 to 0.081) | 0.041 |  |  | | 0.042 (0.003 to 0.081) | | 0.037 |

Note: ^a^ Adjusted for age, left ventricular ejection fraction, creatinine, infarction location and glucose level. Mon2 and FIB are presented after log transformation.

Abbreviations: *CI* confidence interval, *FIB* fibrinogen, *Coef.* coefficients, *Mon2* CD14++CD16+ monocytes.

**sTable 7.** Multivariable Cox proportional hazard models for the relationship between the synergistic role of FIB combined with Mon2 counts and MACEs

|  |  | HR and 95%CI | *P* value |
| --- | --- | --- | --- |
| Fibrinogen stratified by Mon2 | |  |  |
| Low FIB/low Mon2 (reference) | | N. A. | N. A. |
| Low FIB/high Mon2 | | 1.98 (0.86 to 4.55) | 0.106 |
| High FIB/low Mon2 | | 2.38 (1.13 to 4.99) | 0.022 |
| High FIB/high Mon2 | | 4.26 (2.13 to 8.51) | <0.001 |
| Age, year | | 1.08 (0.63 to 1.85) | 0.776 |
| Creatinine, μmol/L | | 0.76 (0.45 to 1.28) | 0.306 |
| Infarct location, anterior wall | | 1.48 (0.88 to 2.49) | 0.135 |
| LVEF, % | | 0.44 (0.25 to 0.77) | 0.004 |
| Glucose, mmol/L | | 1.97 (1.18 to 3.30) | 0.010 |

Abbreviations: *CI* confidence interval, *FIB* fibrinogen, *HR* hazard ratio, *MACEs* major adverse cardiovascular events, *Mon2* CD14++CD16+ monocytes, *LVEF* left ventricular ejection fraction.
